# *Oreocharis jasminina* (Gesneriaceae), a new species from mountain tops of Hainan Island, South China

**DOI:** 10.3897/phytokeys.157.50246

**Published:** 2020-08-26

**Authors:** Shao-Jun Ling, Shu-Ping Guan, Fang Wen, Yu-Min Shui, Ming-Xun Ren

**Affiliations:** 1 Center for Eco-Environmental Restoration Engineering of Hainan Province, College of Ecology and Environment, Hainan University, Haikou 570228, China; 2 College of Life and Pharmaceutical Sciences, Hainan University, Haikou 570228, China; 3 College of Horticulture, Hainan University, Haikou 570228, China; 4 Gesneriad Conservation Centre of China, Guangxi Key Laboratory of Plant Conservation and Restoration Ecology in Karst Terrain, Guangxi Institute of Botany, Guangxi Zhuang Autonomous Region and Chinese Academy of Sciences, Guilin, Guangxi 541006, China; 5 Key Laboratory for Plant Diversity and Biogeography of East Asia, Kunming Institute of Botany, Chinese Academy of Sciences, 650201 Yunnan, Kunming, China

**Keywords:** Hainan Island, new taxon, *
Oreocharis
*

## Abstract

A new species of Gesneriaceae, *Oreocharis jasminina* S.J.Ling, F.Wen & M.X. Ren from Hainan Island, south China, is highlighted and described. The new species is distinguished by its actinomorphic corolla, narrow floral tube and ovate anthers hidden in the floral tube. The new species also showed clear geographic and altitudinal isolation from the three currently-recognised *Oreocharis* species on the Island. Molecular phylogenetic analysis, based on nuclear ITS1/2 and plastid *trn*L-*trn*F sequences, supported the delimitation of the new species, which forms a single lineage with all the other *Oreocharis* species from Hainan Island. The roles of geographic and floral isolation in the evolution of the new species and its affinities are discussed.

## Introduction

The *Oreocharis* Bentham was recently re-circumscribed to a large genus by including ten more genera and over 135 species, based mainly on molecular phylogenetic studies (Möller et al. 2011, 2016; Xu et al. 2017; Möller 2019; Wen et al. 2019). The enlarged genus was predominantly distributed in China with some species in India, Myanmar, Thailand and Vietnam (e.g. Li and Wang 2005; Möller and Clark 2013; Möller et al. 2018). Regardless of the limited differences in habit and fruit structure, *Oreocharis* shows a strikingly-high diversity in floral syndromes (Li and Wang 2005; Wei 2010; Möller and Clark 2013).

As one of the globally-important biodiversity hotspots, Hainan Island harbours about 4000 seed-plant species, of which ca. 500 are endemics (Francisco-Ortega et al. 2010) and which are concentrated in the south-central mountains. Gesneriaceae, in Hainan Island especially, includes a high ratio of species endemism, eight out of the total of 24 species being endemic (Ling et al. 2017a). Currently, three taxa of *Oreocharis* are recorded on Hainan Island and all of them are Hainan-endemic and monophyletic, i.e. *O. dasyantha* Chun, O. dasyantha Chun var. ferruginosa Pan and *O. flavida* Merrill (Li and Wang 2005; Ling et al. 2020), while each of these species shows considerable variations in morphological traits (Wei 2010; Ling et al. 2020a).

During several fieldwork trips in the past three years, we found that some populations of *Oreocharis* on mountain tops in Hainan Island showed obvious differences in various morphological characters. After careful literature studies (Pan 1987; Li and Wang 2005; Wei 2010) and morphological and molecular examinations, we are convinced that populations from the mountain tops of Mt. Yingge and Mt. Limu represent a new species, which we report and describe here.

## Materials and methods

### Morphological observations

The field study and conservation on Gesneriaceae were undertaken by two of the authors (SJL and MXR) over a long period of time, especially focusing on the Hainan-endemic species (Ling et al. 2017a, b; Xing et al. 2018; Li et al. 2019). Morphological observations and measurements were carried out, based on living plants during fieldwork. All available specimens of *Oreocharis* species, stored in the herbaria in China (PE, KUN, IBK and IBSC), were examined. We also downloaded all *Oreocharis* specimens from JSTOR Global Plants (http://plants.jstor.org), and Chinese Virtual Herbarium (http://www.cvh.ac.cn) to compare detailed morphological traits between the proposed new species with the currently-accepted species of *Oreocharis*. Specifically, we compared morphological traits of the possible new species with all the three currently-recognised *Oreocharis* species from Hainan Island, i.e. *O. dasyantha*, O. dasyantha var. ferruginosa and *O. flavida*. The specimens of new species were collected over the past two years and deposited in the herbarium of Hainan University (HUTB) and Kunming Institute of Botany, Chinese Academy of Sciences (KUN).

### Taxonomic sampling, DNA extraction and molecular markers

The leaf samples of *O. dasyantha*, O. dasyantha var. ferruginosa, *O. flavida* and the putative new species were collected in the field and dried in a vascular bag with silica gel. Total genomic DNA extraction was conducted using CTAB methods (Doyle and Doyle 1987). One nuclear ribosomal DNA (nrDNA) sequence, the ITS region comprising spacer 1, the 5.8S gene and spacer 2 (White et al. 1990) and one chloroplast DNA (cpDNA) intron-spacer region *trn*L-*trn*F (Taberlet et al. 1991) were used in this study. Laboratory procedures followed Ling et al. (2020) and newly-acquired sequences were deposited in GenBank (Table 2).

### Alignments and phylogenetic analyses

According to Möller et al. (2011), Chen et al. (2014) and Ling et al. (2020), *Oreocharis sinohenryi* (Chun) Mich.Möller & A.Weber which had the closest phylogenetic relationships with the Hainan *Oreocharis* taxa was used as outgroup with sequences (Genbank with accession numbers HQ632913 and HQ633009). The original chromatograms from both directions of the ITS1/2 and *trn*L-*trn*F sequences were evaluated using Bioedit (Hall 1999) for base confirmation and contiguous sequences editing, then we manually aligned sequences, where necessary, using MEGA v.6.5 (Kumar et al. 2008) and ambiguous positions were excluded from the alignments. The ITS1/2 and *trn*L-*trn*F were concatenated to a single matrix after a congruency test by PAUP* 4.0a164 (Swofford 2003). Bayesian Inference (BI) analysis was conducted using MrBayes version 3.1.2 (Huelsenbeck and Ronquist 2001) and Maximum Likelihood (ML) analysis was performed using MEGA v.6.5 (Kumar et al. 2008). Both procedures followed the Ling et al. (2020), based on the combined ITS1/2 and *trn*L-*trn*F sequences.

## Results

### Phylogenetic reconstruction

The combined ITS1/2 and *trn*L-*trn*F datasets were 640 and 818 bp long, amongst which 64 and 17 were polymorphic sites and 27 and 6 were parsimony-informative sites, respectively. The aligned dataset was 1458 bp long and a total number of 81 polymorphic sites were measured, of which 33 were parsimony-informative sites. There was no significant incongruence, based on the incongruence length difference (ILD) test between the ITS1/2 and *trn*L-*trn*F (p > 0.05).

**Figure 1.**
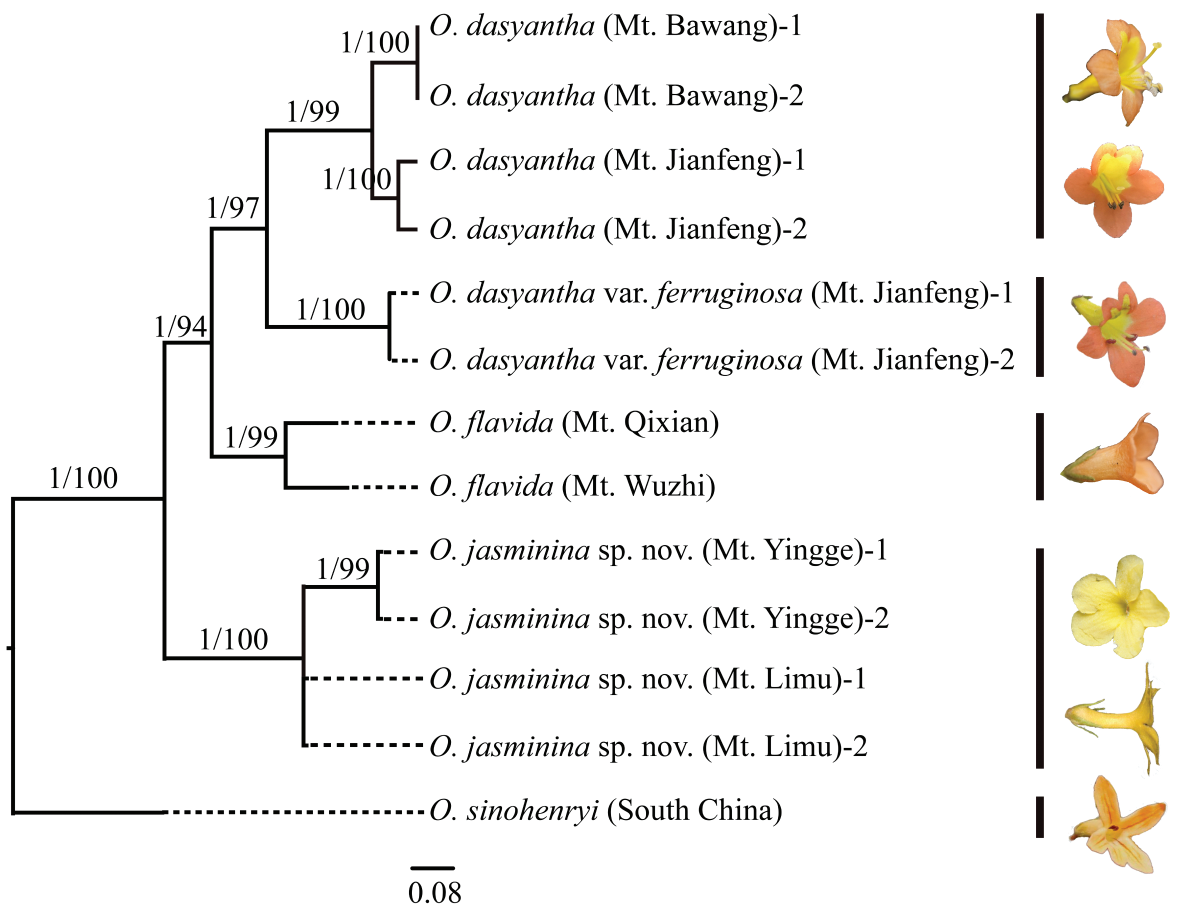
Molecular phylogeny of Hainan *Oreocharis* taxa with outgroup *O. sinohenryi*, based on the combined nuclear ribosomal DNA (nrDNA) sequence ITS1/2 and chroloplast gene *trn*L-*trn*F data matrices. Posterior probability (PP) and Bootstrap value (BS) are showed above branches.

Molecular phylogeny recognised the individuals from different mountains and these were grouped as separate lineages. The putative new species from Mt. Limu and Mt. Yingge is accepted as a new species with PP (posterior probability) = 1 and BS (bootstrap value) = 100% (Fig. 1). All the *Oreocharis* species from Hainan Island form a single lineage with relatively-high support (Fig. 1).

### Taxonomic treatment

#### Oreocharis jasminina

Taxon classificationPlantaeLamialesGesneriaceae

S.J.Ling, F.Wen & M.X. Ren sp.nov.

964314ED-2817-544D-80C7-534A26EF51D5

urn:lsid:ipni.org:names:77211189-1

Figs 2 , 3

##### Diagnosis.

*Oreocharis jasminina* has the closest phylogenetic relationship with *O. dasyantha*, O. dasyantha var. ferruginosa and *O. flavida* with very high support values, all being Hainan-endemic and monophyletic. *O. jasminina* can be easily distinguished from them by having: (1) a long and narrow floral tube (both *O. dasyantha* and O. dasyantha var. ferruginosa have conical floral tubes, *O. flavida* has campanulate-tubular floral tube); (2) yellow and actinomorphic corolla (both *O. dasyantha* and O. dasyantha var. ferruginosa are zygomorphic with orange-red to yellow corolla, *O. flavida* is actinomorphic with orange corolla); (3) didynamous stamens with ovate anthers hidden in the floral tube (both *O. dasyantha* and O. dasyantha var. ferruginosa have exposed didynamous stamens with ovate anthers, *O. flavida* has four equivalent stamens with horseshoe-shaped anthers included in the floral tube) (Table 1, Fig. 4).

**Figure 2.**
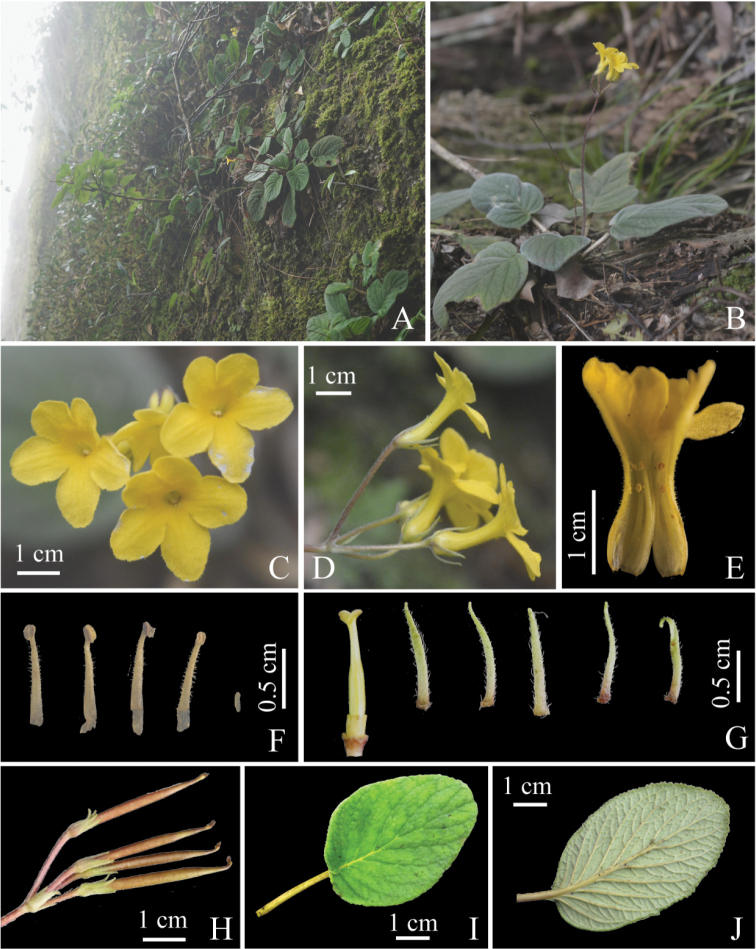
*Oreocharis jasminina* sp. nov. **A** Habitat **B** habit **C** face view of corolla **D** lateral view of corolla **E** opening flower showing stamens and staminode **F** stamens and staminode **G** pistil and sepals **H** fruit pods **I** adaxial leaf surface **J** abaxial leaf surface.

**Figure 3.**
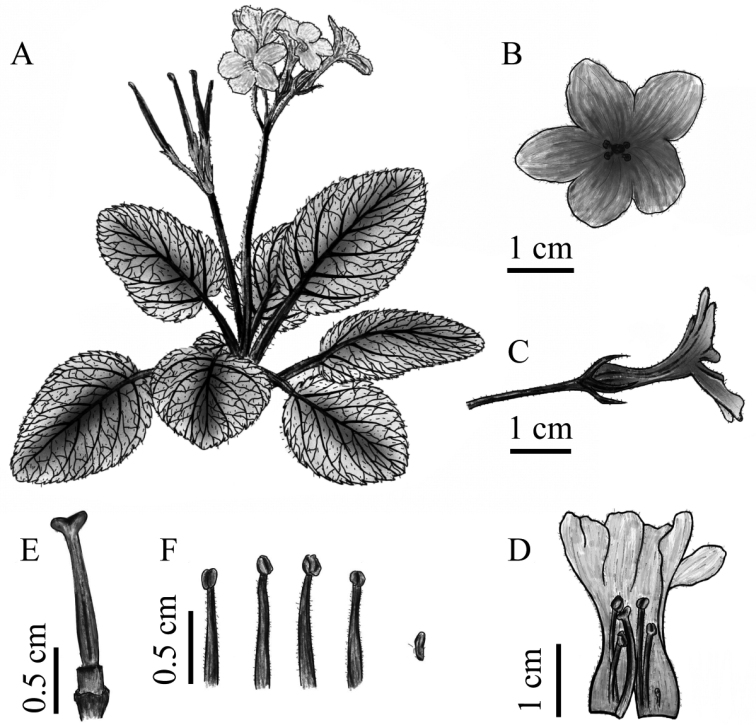
*Oreocharis jasminina* sp. nov. (all drawings based on the holotype *S.J.Ling 20181126*–*01* in HUTB, drawn by S.P. Guan). **A** Habit **B** face view of corolla **C** lateral view of corolla **D** opening corolla showing pistil and stamens **E** pistil **F** stamens and staminode.

**Figure 4.**
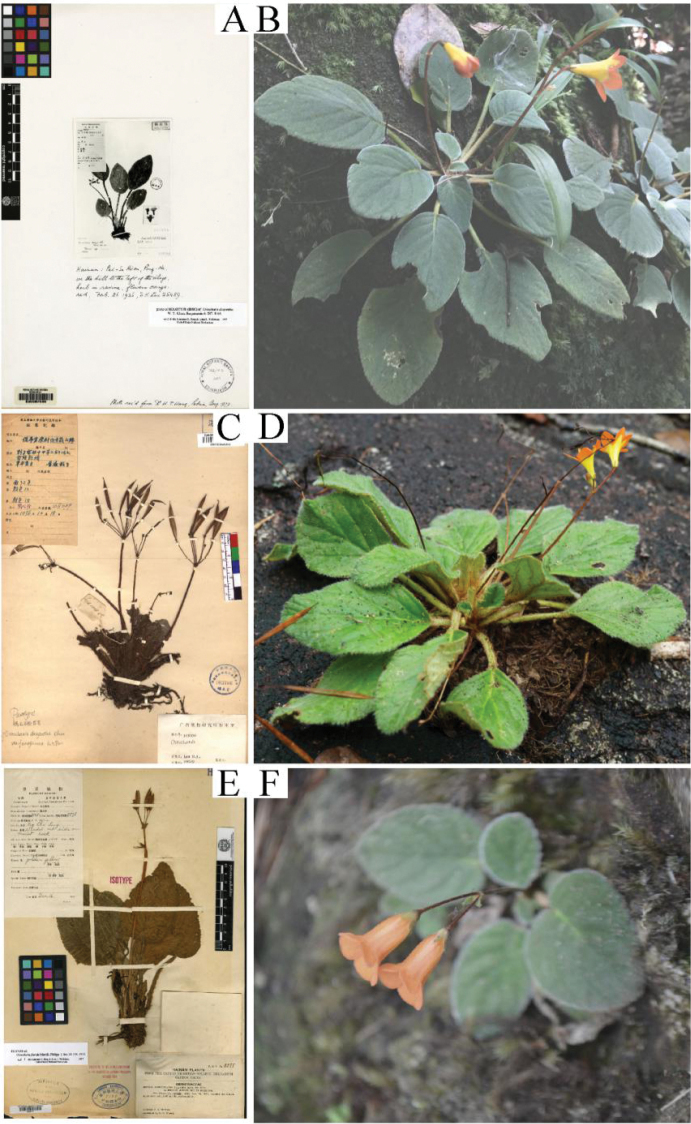
Three formerly-recognised *Oreocharis* taxa in Hainan Island. *Oreocharis dasyantha* (**A, B**), Oreocharis dasyantha var. ferruginosa (**C, D**) and *Oreocharis flavida* (**E, F**).

##### Type.

China. Hainan: Qiongzhong County (琼中县), Limu Mountain, 1350 m a.s.l., on moist rocks, 26 Nov 2018, *S.J.Ling 2018112601* (holotype: HUTB!; Isotypes: HUTB!, KUN!).

##### Description.

Perennial herb, rhizomatous, leaves basal; 4.0–10.0 cm long, 2–3 mm in diameter, densely pale brown villous or woolly; leaf blade ovate to broadly ovate, rarely elliptic or obovate, 6–11 × 4–8 cm, adaxially densely grey to brown pubescent, abaxially sparsely to densely grey or grey-brown pubescent, sparsely brown villous along veins which are adaxially sunken and abaxially ridged, lateral veins 6–7 on each side of midrib, base often cordate to rounded, margin nearly entire to shallowly crenate, apex rounded. Cymes axillary, 2–3, inflorescence 3–10-flowered; Peduncle 9–16 cm long, sparsely pale grey villous; bracts 2, linear to narrowly triangular, outside densely villous, apex acuminate, cuneate to triangular, margin entire; pedicel 1.5–2.2 cm long, densely pale brown villous to woolly. Calyx 5-lobed, divided to base, lobes green, narrowly lanceolate, 9–11 × ca. 2 mm, apex acuminate, margin entire, outside villous, inside glabrous. Corolla yellow, 1.7–2.2 cm long, outside pubescent; tube thin tubular, 1.8–2.1 cm × 3–4.5 mm, limb barely 2-lipped, adaxial lip shallowly 2-lobed from near base, abaxial 3-lobed slightly equal. Stamens 4, 8–9 mm long, included, adnate to corolla 4–5 mm from base; filaments slender, pubescent; anthers ovate, 2-loculed, dehiscing transversely; staminode 1, adnate to corolla 2–4 mm from base, ca. 2 mm. Disc ca. 1 mm high, entire. Pistil ca. 7 mm long; ovary cylindrical, ca. 5 mm long, glabrous. Stigma 2, equal, suborbicular. Capsula linear, 3–4 cm long, glabrous to sparsely puberulent.

**Table 1.** Diagnostic morphological characters of *Oreocharis jasminina* sp. nov. and all the three currently-recognised species in Hainan Island.

Characters	*Oreocharis jasminina* sp. nov.	*O. dasyantha*	O. dasyantha var. ferruginosa	*O. flavida*
Corolla colour	yellow	orange-red to yellow	orange-red to yellow	orange
Corolla tube	narrowly tubular,1.7–2.2 cm long 1.8–2.2 cm × 3–4.5 mm	conical,1.6–2.4 cm long 0.9–2 cm × 6–7 mm	conical, ca.1.6 cm, tube 9–1.1 mm	campanulate-tubular,1.7–1.9 cm long 1.6–1.8 cm × 6–8 mm
Corolla symmetry	actinomorphic	zygomorphic	zygomorphic	actinomorphic
Leaf blade shape	ovate to broadly ovate, rarely elliptic or obovate	ovate-elliptic to broadly ovate	ovate-elliptic to broadly ovate	ovate-elliptic to broadly ovate, rarely broadly elliptic
Leaf base shape	cordate to rounded	oblique, cuneate to subrounded or cordate	sometimes oblique, cuneate to subcordate	oblique, subrounded
Leaf base margin	nearly entire to shallowly crenate, apex rounded	serrulate or crenate-serrate, apex acute to rounded	crenate-serrate	shallow crenate
Stamens	included, didynamous, staminode 1	exposed, didynamous, staminode absent	exposed, equivalent, staminode absent	included, equivalent, staminode 1
Anthers	ovate, 2-loculed, dehiscing transversely	broadly oblong, 2-loculed, dehiscing longitudinally	broadly oblong, 2-loculed, dehiscing longitudinally	horseshoe-shaped,1-loculed, dehiscing transversely
Filaments	pubescent	pubescent	pubescent	glabrous
Pistil	ca. 9 mm long	ca. 22mm long	ca. 22mm long	ca.9 mm long

##### Phenology.

*Oreocharis jasminina* flowers from September to December and fruits from November to January.

##### Distribution and habitat.

*Oreocharis jasminina* is currently only found in cloud forests on the mountain tops of Mt. Limu and Mt. Yingge, in the middle of Hainan Island. The habitat of *O. jasminina* is on the moss layer on wet rocks under cloud forests.

##### Etymology.

The specific epithet refers to the yellow and narrowly tubular corolla of this new species.

##### Vernacular name.

迎春花马铃苣苔 (Yíng Chūn Huā Mǎ Líng Jù Tái) is the Chinese name for *Oreocharis jasminina*, the first three characters meaning ‘winter jasmine’, indicating its similar floral syndromes to *Jasminum nudiflorum* Lindl. The last four characters are the Chinese name for *Oreocharis*.

##### Conservation status.

*Oreocharis jasminina* is, so far, known only from the two locations with about 800–1000 individuals. The populations are under threat due to the restricted and fragmented habitat. Therefore, we propose that *O. jasminina* should be considered as ‘Vulnerable’ (VU), according to the IUCN Red List Categories and Criteria (IUCN 2012).

**Table 2.** List of Hainan *Oreocharis* taxa and outgroup *O. sinohenryi* used in the phylogenetic analysis, including respective Genbank accession and voucher numbers.

Species	*trn*L-*trn*F	ITS1/2	Voucher Number
*O. dasyantha* Chun (Mt. Bawang)-1	MK587993	MK587954	S.J.Ling & M.X. Ren 2015011803 (HUTB)
*O. dasyantha* Chun (Mt. Bawang)-2	MK587994	MK587954	S.J.Ling & M.X. Ren 2015011804 (HUTB)
*O. dasyantha* Chun (Mt. Jianfeng)-1	MK587995	MK587955	S.J.Ling 2015102201 (HUTB)
*O. dasyantha* Chun (Mt. Jianfeng)-2	MK587996	MK587955	S.J.Ling 2015102202 (HUTB)
O. dasyantha Chun var. ferruginosa Pan (Mt. Jianfeng)-1	MK587954	MK587956	S.J.Ling 2015102203 (HUTB)
O. dasyantha Chun var. ferruginosa Pan (Mt. Jianfeng)-2	MK587954	MK587957	S.J.Ling 2015102204 (HUTB)
*O. flavida* Merrill (Mt. Qixian)	MK587947	MK587990	S.J.Ling 2018112901 (HUTB)
*O. flavida* Merrill (Mt. Wuzhi)	MK587989	MK587943	S.J.Ling 2018112902 (HUTB)
*O. jasminina* (Mt. Yingge)-1	MK587987	MK587948	S.J.Ling 2018112601 (HUTB)
*O. jasminina* (Mt. Yingge)-2	MK587988	MK587950	S.J.Ling 2018112602 (HUTB)
*O. jasminina* (Mt. Limu)-1	MK587981	MK587949	S.J.Ling 2018112603 (HUTB)
*O. jasminina* (Mt. Limu)-2	MK587982	MK587953	S.J.Ling 2018112604 (HUTB)
*O. sinohenryi* (Chun) Mich.Möller & A.Weber	HQ632913	HQ633009	M.Möller MMO 07-1150 (E)

### Key to *Oreocharis jasminina* and its closely-related and sympatric species in Hainan Island

**Table:** 

1	Anthers horseshoe-shaped, 1-loculed, dehiscing transversely	***O. flavida***
–	Anthers broadly oblong, 2-loculed, dehiscing longitudinally	**2**
2	Stamens included, floral tube thin tubular, corolla yellow	***O. jasminina***
–	Stamens exposed, floral tube conical, corolla orange-red	**3**
3	Leaf blade adaxially grey pubescent, base oblique, subrounded to cordate, margin serrulate; petiole to 14.5 cm, densely pale brown villous; cymes 1–3(or 4)-flowered; corolla 1.7–2.4 cm, tube 1.1–2 cm	***O. dasyantha***
–	Leaf blade adaxially grey to brown pubescent and villous, base sometimes oblique, cuneate to subcordate, margin crenate-serrate; petiole to 6 cm, densely pale brown woolly; cymes 3–8-flowered; corolla ca. 1.6 cm, tube 9–11 mm	**O. dasyantha var. ferruginosa**

## Discussion

Our former study showed the new species *O. jasminina* and the three other Hainan-endemic taxa are homologous, indicating these species in Hainan Island had a common origin (Ling et al. 2020). The new species is only found on mountain tops higher than 1200 m in two mountains, Mt. Limu and Mt. Yingge, located at the middle of Hainan Island. These mountain tops likely formed island-like habitats because the deep and wide valleys interrupted gene flows, resulting in population differentiation and speciation (Shen et al. 2017; Ling et al. 2017a, b; Xing et al. 2018). Such ‘sky islands’ may be the main reason for the origin and maintenance of this Hainan-endemic alpine species (Robin et al. 2015; Ling et al. 2017a).

**Figure 5.**
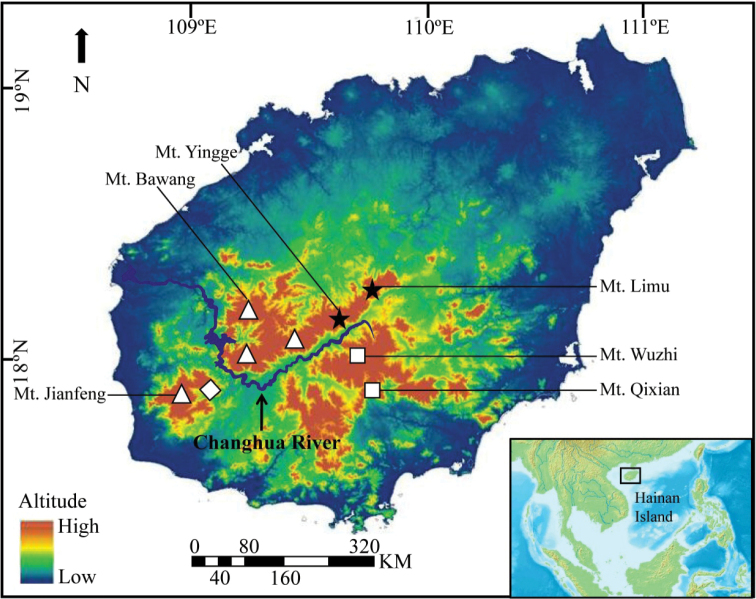
Geographic distribution of *Oreocharis jasminina* sp. nov. and the three congeners on Hainan Island. ★ *O. jasminina* sp. nov. ∆ *O. dasyantha* ◊ O. dasyantha var. ferruginosa □ *O. flavida*.

The new species also shows a clear geographic isolation from the three currently-recognised *Oreocharis* taxa on Hainan Island. The new species *O. jasminina* was only found in Mt. Limu and Mt. Yingge in the middle of the island, while *O. dasyantha* and O. dasyantha var. ferruginosa are restricted to the west side of the Island and *O. flavida* was only found in the east side (Fig. 5). They are isolated by a large river, the Changhua River (the second largest river on Hainan Island). Li et al. (2019) found that the geographic isolation by the Changhua River is a driving force for the great population differentiation in the two Hainan-endemic Gesneriaceae species, *Primulina heterotricha* (Merr.) Yan Liu and *Metapetrocosmea peltata* (Merr. et Chun) W. T. Wang. Thus, the geographic isolation by rivers or valleys may also play a key role in the evolution of *O. jasminina* and other Hainan-endemic *Oreocharis* taxa. However, the relative contributions of such geographic isolation and altitudinal differentiation are still in need of further experimental examination.

Floral symmetry is widely recognised as a key trait in pollination and taxonomy. Normally, the zygomorphic corolla possesses higher pollen-transfer efficiency than the actinomorphic corolla (Sargent 2004). *Oreocharis jasminina* has yellow actinomorphic corolla with a long and narrow floral tube, differing from *O. dasyantha* and O. dasyantha var. ferruginosa (both have zygomorphic corolla). Although *O. flavida* has an actinomorphic flower, its campanulate corolla with four equivalent stamens and horseshoe-shaped anthers make it distinct from the new species *O. jasminina* (Table 1).

Floral shape was expected to be a vital factor in generating floral isolation and evolutionary shifts (Castellanos et al. 2004; Muchhala 2007). Generally, the floral shape has a strong connection with the expected pollinators in Gesneriaceae, for example, bees or hummingbirds for tubular flowers, bats for campanulate flowers and subcampanulate flowers having generalised pollination systems (Martén-Rodríguez et al. 2009). *O. jasminina* has thin-tubular corolla (Fig. 1), differing from *O. dasyantha*, O. dasyantha var. ferruginosa (both are conical corolla) and *O. flavida* (campanulate-tubular corolla), indicating a possible pollination mechanism associated with the long-tongued butterflies and moths. Such distinctive morphological differences indicate different pollination adaptation and clear reproductive isolation amongst these taxa, suggesting *O. jasminina* should be treated as a new species.

## Supplementary Material

XML Treatment for Oreocharis jasminina

## Appendix 1

**Table:** The *Oreocharis* specimens we checked in this study.

Voucher Number	Species	Voucher Number	Species
PE00030859	*Oreocharis rhytidophylla*	bm000041491	*Oreocharis aurantiaca*
IBSC0004917	*Oreocharis henryana*	gh00353683	*Oreocharis dentata*
A00353713	*Oreocharis magnidens*	k000858130	*Oreocharis maximowiczii*
bm000041734	*Oreocharis benthamii*	e00087519	*Oreocharis tubicella*
e00067455	*Oreocharis cavaleriei*	e00087520	*Oreocharis nemoralis*
a00025113	*Oreocharis flavida*	IBSC0004912	*Oreocharis aurea*
bm000041721	*Oreocharis georgei*	p04060117	*Oreocharis forrestii*
KUN1219176	*Oreocharis cordato-ovata*	e00135096	*Oreocharis amabilis*
e00087535	*Oreocharis dasyantha*	e00135074	*Oreocharis bodinieri*
bm000041708	*Oreocharis cinnamomea*	gh00353695	Oreocharis benthamii var. reticulata
PE00030854	*Oreocharis tubiflora*	p04060171	*Oreocharis delavayi*
IBSC0004920	*Oreocharis xiangguiensis*	CSFI028502	*Oreocharis brachypodus*
PE19401111	*Oreocharis amabilis*	PE02052999	*Oreocharis heterandra*
IBSC0550960	*Oreocharis sericea*	IBSC0550860	*Oreocharis cordatula*
IBSC0550891	*Oreocharis georgei*	IBSC0550875	*Oreocharis elliptica*
GZTM0075588	*Oreocharis primuloides*	JIU63907	*Oreocharis speciosa*
PE02052990	Oreocharis argyreia var. angustifolia	PE02053568	*Oreocharis eximia*
IBK00054784	*Oreocahris auricula*	PE01909883	*Oreocharis mileensis*
KUN1219104	*Oreocharis hekouensis*	WUK0494363	*Oreocharis saxatilis*
PE02053062	*Oreocharis concava*	PE01486523	*Oreocharis rosthornii*
PE02106072	*Oreocharis begoniifolia*	KUN1385365	*Oreocharis nanchuanica*
KUN1385575	*Oreocharis urceolata*	HITBC106680	*Oreocharis longifolia*
PE00030861	*Oreocharis rotundifolia*	IBSC0548683	*Oreocharis chienii*
PE02053433	*Oreocharis acaulis*	KUN1385156	*Oreocharis bullata*
PE02241281	*Oreocharis burttii*	PE02053072	*Oreocharis cinerea*
KUN1220227	*Oreocharis convexa*	IBK00054466	*Oreocharis cotinifolia*
PE00155697	*Oreocharis craibii*	WUK0350789	*Oreocharis crenata*
IBSC0550709	*Oreocharis dalzielii*	PE02106079	*Oreocharis dinghushanensis*
IBSC0551649	*Oreocharis esquirolii*	PE02052984	*Oreocharis fargesii*
IBSC0649611	*Oreocharis flabellata*	PE02053533	*Oreocharis gamosepala*
PE02052812	*Oreocharis giraldii*	PE02106025	*Oreocharis glandulosa*
PE02052995	*Oreocharis humilis*	PE01548041	*Oreocharis jiangxiensis*
PE02021009	*Oreocharis lancifolia*	FJSI004239	*Oreocharis leiophylla*
PE02053066	*Oreocharis leucantha*	IBSC0550069	*Oreocharis lungshengensis*
IBSC0551655	*Oreocharis mairei*	PE02053564	*Oreocharis minor*
WUK0160594	*Oreocharis muscicola*	IBSC0548476	*Oreocharis notochlaena*
PE02106041	*Oreocharis obliqua*	PE02052801	*Oreocharis obliquifolia*
PE02088092	*Oreocharis obtusidentata*	PE02053064	*Oreocharis pankaiyuae*
PE01270485	*Oreocharis primuloides*	WUK0213194	*Oreocharis pumila*
PE02053576	*Oreocharis pinnatilobata*	KUN1241303	*Oreocharis primuliflora*
PE02053532	*Oreocharis rhombifolia*	PE00030693	*Oreocharis ronganensis*
PE00030747	*Oreocharis sichuanensis*	IBSC0550081	*Oreocharis sichuanica*
IBK00054319	*Oreocharis sinensis*	IBK00207093	*Oreocharis sinohenryi*
PE02053579	*Oreocharis stenosiphon*	IBSC0548730	*Oreocharis stewardii*
PE02053570	*Oreocharis trichantha*	HEAC0016525	*Oreocharis villosa*
PE02053561	*Oreocharis wangwentsaii*	PE02053077	*Oreocharis wanshanensis*
Y. M. Shui et al. B2014-299 (KUN)	*Oreocharis synergia*	Y.M.Shui et al. N699 (KUN)	*Oreocharis ninglangensis*
PE-02114626	*Oreocharis duyunensis*	IBSC0825078	*Oreocahris ovata*
KUN1219115	*Oreocharis acutiloba*	PE00030682	*Oreocharis agnesiae*
PE00140281	*Oreocharis billburttii*	PE02025205	*Oreocharis elegantissima*
IBSC0649550	*Oreocharis latisepala*	PE00030685	*Oreocharis parva*
Z.K. Wu et al.C2016055 (KUN)	*Oreocharis parvifolia*	PE02025202	*Oreocharis pinfaensis*
IBSC0548691	*Oreocharis shweliensis*	PE01909893	*Oreocharis tongtchouanensis*
Y.M.Shui, Y.K.Sima & W.H.Chen B2013-258 (KUN)	*Oreocharis crispata*	Y.M.Shui et al. 91309 (KUN)	*Oreocharis jinpingensis*
Bo Pan & M. Q. Han HMQ859 (IBK)	*Oreocharis purpurata*	Yun-Hong Tan 3308 (HITBC)	*Oreocharis tsaii*
Averyanov, L., Hiep, N.T., Khang, N.S., Thang, N.D. & Qui, L.D. CPC 7019 (KUN)	*Oreocharis blepharophylla*	Jia-Mei Li and Yao-Guang Zhang 1606151 (HEAC)	*Oreocharis zhenpingensis*
Bo Pan & Jia-Jia Wei et al. GY002 (IBK)	*Oreocharis curvituba*	C.Z. Yang et al. 35042620140913001 (FNU)	*Oreocharis striata*
Y.M. Shui et al. B2013-551 (KUN)	*Oreocharis longituba*	Averyanov, L., Hiep, N.T., Khang, N.S., Thang, N.D. & Qui, L.D. CPC 7175 (KUN)	*Oreocharis argyrophylla*
Y.M. Shui et al. B2013-550	*Oreocharis grandiflora*	T.V. Do 57 (VNMN)	*Oreocharis caobangensis*
L.H. Yang et al. YLH197 (IBSC)	*Oreocharis pilosopetiolata*	Li-Hua Yang et al. YLH285 (IBSC)	*Oreocharis uniflora*
Ying Guo C2015005 (KUN)	*Oreocharis panzhouensis*	L. E. Yang 60 (KUN)	*Oreocharis rubrostriata*
Yan Liu and Wei-Bin Xu 08018 (IBK)	*Oreocharis dayaoshanioides*	Yun-Hong Tan 6925 (HITBC)	*Oreocharis glandulosa*
PE 02053063	*Oreocharis farreri*	Lin Qin-Wen et al. 0016 (FAFU)	*Oreocharis baolianis*
IBSC0548624	*Oreocharis guileana*	IBK00054993	Oreocharis dasyantha var. ferruginosa
